# Testing Separability and Independence of Perceptual Dimensions with General Recognition Theory: A Tutorial and New R Package (*grtools*)

**DOI:** 10.3389/fpsyg.2017.00696

**Published:** 2017-05-23

**Authors:** Fabian A. Soto, Emily Zheng, Johnny Fonseca, F. Gregory Ashby

**Affiliations:** ^1^Department of Psychology, Florida International University, MiamiFL, USA; ^2^Department of Statistics and Applied Probability, University of California, Santa Barbara, Santa BarbaraCA, USA; ^3^Department of Mathematics and Statistics, Florida International University, MiamiFL, USA; ^4^Department of Psychological and Brain Sciences, University of California, Santa Barbara, Santa BarbaraCA, USA

**Keywords:** general recognition theory, R package, perceptual separability, perceptual independence, Garner interference, identification task, decisional separability, signal detection theory

## Abstract

Determining whether perceptual properties are processed independently is an important goal in perceptual science, and tools to test independence should be widely available to experimental researchers. The best analytical tools to test for perceptual independence are provided by General Recognition Theory (GRT), a multidimensional extension of signal detection theory. Unfortunately, there is currently a lack of software implementing GRT analyses that is ready-to-use by experimental psychologists and neuroscientists with little training in computational modeling. This paper presents *grtools*, an R package developed with the explicit aim of providing experimentalists with the ability to perform full GRT analyses using only a couple of command lines. We describe the software and provide a practical tutorial on how to perform each of the analyses available in *grtools*. We also provide advice to researchers on best practices for experimental design and interpretation of results when applying GRT and *grtools*

## Introduction

In perceptual science, an important amount of effort has been dedicated to understanding what aspects of stimuli are represented independently (e.g., Bruce and Young, 1986; Ungerleider and Haxby, 1994; Haxby et al., 2000; Kanwisher, 2000; Vogels et al., 2001; Stankiewicz, 2002; Op de Beeck et al., 2008). Independent processing of two stimulus properties is interesting, because it implies that those properties are given priority by the perceptual system, which allocates a specific set of resources to the processing of each. On the other hand, some properties are said to be processed “holistically” or “configurally,” which is equivalent to saying that they cannot be processed independently (e.g., Thomas, 2001; Richler et al., 2008; Mestry et al., 2012). Such holistic processing is also important to understand perception (see Farah et al., 1998; Maurer et al., 2002; Richler et al., 2012). In sum, determining whether perceptual properties are processed independently is an important goal in perception science, and tools to test independence should be widely available to experimental researchers.

Currently, the best analytical tools to test for independence of perceptual processing are provided by General Recognition Theory (GRT; Ashby and Townsend, 1986; for a tutorial review, see Ashby and Soto, 2015). GRT is an extension of signal detection theory to cases in which stimuli vary along two or more dimensions. It offers a framework in which different types of dimensional interactions can be defined formally and studied, while inheriting from signal detection theory the ability to dissociate perceptual from decisional sources for such interactions. Still, the implementation of GRT analyses requires an important amount of technical knowledge, so this task has been almost exclusively performed by computational modelers. The lack of software implementing GRT analyses that is ready-to-use by untrained researchers has probably reduced the application of GRT among experimental psychologists and neuroscientists.

This paper presents *grtools*, an R package developed with the explicit aim of providing experimentalists with the ability to perform full GRT analyses using only a couple of command lines. We describe the software and provide a practical tutorial on how to perform each of the analyses available in *grtools*. The goal is to give a step-by-step guide for researchers interested in applying GRT to their own research problems. Readers interested in the theory behind these analyses should consult the recent review by Ashby and Soto (2015) and the papers referenced therein.

## General Recognition Theory

**FIGURE 1:**
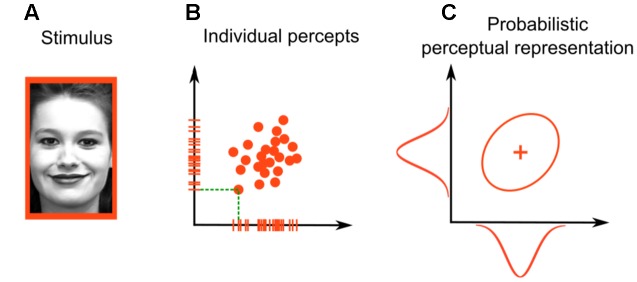
**Stimulus representation in general recognition theory**. For **(A)** faces varying in gender (male or female) and emotional expression (sad or happy), the representation is two-dimensional. Each stimulus presentation produces **(B)** a point in the two-dimensional space. The whole distribution of such perceptual effects can be described through **(C)** a two-dimensional probability distribution. Face photograph in **(A)** obtained from the Extended Cohn-Kanade (CK+) database (S74, Jeffrey Cohn; Kanade et al., 2000; Lucey et al., 2010), consent for publication obtained by the original authors.

General recognition theory is a multivariate extension of signal detection theory to cases in which stimuli vary on more than one dimension (Ashby and Townsend, 1986; for a review, see Ashby and Soto, 2015). As in signal detection theory, GRT assumes that different presentations of the same stimulus produce slightly different perceptual representations. However, because stimuli vary in multiple dimensions, perceptual representations vary along multiple dimensions at the same time. For example, imagine that you are interested in the dimensions of gender (male vs. female) and emotional expression (neutral vs. sad) in faces. A single presentation of a happy female (**Figure 1A**) would produce a perceptual effect—a single point in the two-dimensional space of perceived gender and emotional expression. **Figure 1B** shows an example of such a two-dimensional perceptual space; each point in the figure represents a percept evoked by a single stimulus presentation, and the tick marks on the axes represent the corresponding percept values on both perceptual dimensions. The green dotted lines show this correspondence between points and tick marks more clearly for a single perceptual effect.

Thus, the representation of a happy female face in **Figure 1** is probabilistic across trials and can be summarized through a probability distribution. Assuming that this distribution is a two-dimensional Gaussian (a common assumption in GRT, as well as in signal detection theory and many other statistical models), we can represent it graphically by the ellipse in **Figure 1C**, which is the shape of the cloud of points that are produced by the presented face. The plus sign inside the ellipse represents the mean of the distribution. The bell-shaped unidimensional Gaussians plotted on the axes of **Figure 1C** represent the distribution of tick marks along a single dimension. These are called *marginal distributions*.

### Forms of Independence Defined within GRT

**FIGURE 2:**
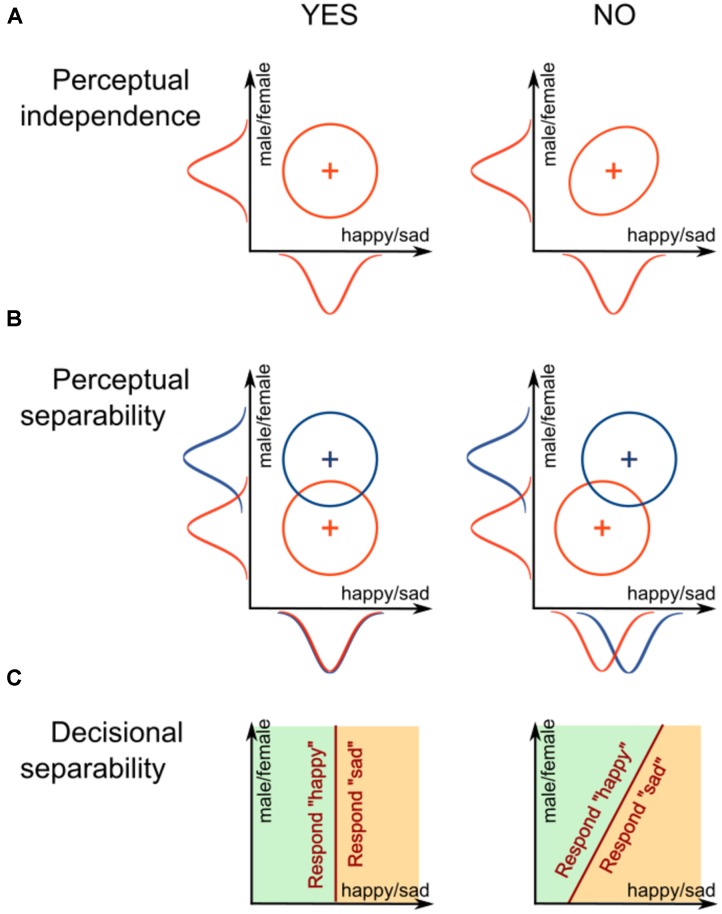
**Schematic representation of different forms of interaction between dimensions defined in general recognition theory: (A)** perceptual independence, **(B)** perceptual separability, and **(C)** decisional separability.

**Figure 2A** shows two distributions that are equivalent except for the correlation between the values of percepts on the two dimensions. The distribution to the left shows no correlation; this is a case of *perceptual independence* (PI), where the perceived value of expression does not depend on the perceived value of gender. The distribution to the right shows a positive correlation, which is an example of a *failure of PI.* In this case, whenever the face is presented, the more it is perceived as female-looking, the more likely it will also be perceived as more sad. It is important to note that PI is defined for a single stimulus.

Another important concept in GRT is related to how two or more stimuli are perceived. Each stimulus with a unique combination of gender and emotional expression can be represented by its own probability distribution. For example, **Figure 2B** shows perceptual distributions for two happy faces that differ in their gender. Note how the distributions in the left panel are aligned on the *x*-axis, representing expression, and how their marginal distributions overlap. This is a case of *perceptual separability* (PS) of emotional expression from gender; the perception of happiness is not affected by a change in gender. The right panel of **Figure 2B** shows an example of the opposite case, a *failure of PS*. In this case, the two distributions do not align and their marginal distributions do not overlap. The male face is perceived as more “happy” than the female face.

In a typical behavioral experiment, participants would be asked to identify something about the presented stimulus, like a face’s expression, gender, or both. According to GRT, participants achieve this by dividing the perceptual space into response regions. For example, a participant asked to report gender might divide the perceptual space as shown in **Figure 2C**, using a linear bound. When a percept lands in the left area, the participant reports “happy,” and when the percept lands in the right area, the participant reports “sad.” Note how the decision bound in the left panel of **Figure 2C**, which is orthogonal to the expression dimension, divides the space in the same way across all values of gender. This is a case of *decisional separability* (DS) of emotional expression from gender; the decisions about expression are not affected by the face’s gender. The right panel of **Figure 2C** shows an example of the opposite case, a *failure of DS.* In this case, the bound is tilted instead of orthogonal, and the area given to the “happy” response is much smaller in the “male” end of the identity dimension than in the “female” end. That is, the observer is biased to answer “happy” more often for the female faces than for male faces.

### The 2 × 2 Identification Task

The most widely used task to study the independence of stimulus dimensions using GRT is the 2 × 2 identification task. On each trial of an identification task, a stimulus is presented and it must be identified by pressing a specific response button. Each stimulus must have a value on at least two dimensions or features (that we want to test for independence), A and B. If there are only two values per dimension, we obtain the 2 × 2 design with stimuli A_1_B_1_, A_1_B_2_, A_2_B_1_, and A_2_B_2_.

**FIGURE 3:**
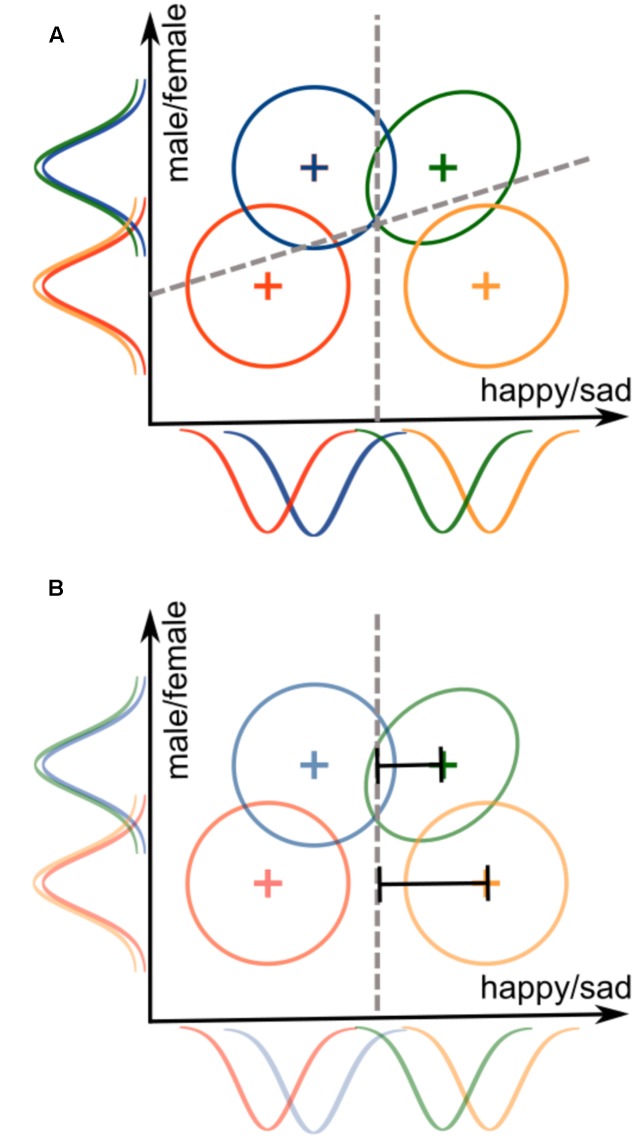
**Illustration of a General Recognition Theory (GRT) model for the (A)** 2 × 2 identification task and the **(B)** 2 × 2 Garner filtering task. Both models use the exact same stimulus representations, but the decision bounds represent the different demands of the tasks. The identification task requires dividing the space into four response regions, one for each stimulus, whereas the Garner filtering task requires dividing the space into two regions, one for each level of the relevant dimension.

For example, consider a 2 × 2 face identification experiment where the two varying dimensions are face emotional expression (dimension A) and gender (dimension B). Assume that the levels for the emotion dimension are happy (A_1_) and sad (A_2_), whereas the levels for the gender dimension are male (B_1_) and female (B_2_). Thus, a 2 × 2 identification task would create four face stimuli: happy-male (A_1_B_1_), sad-male (A_1_B_2_), happy-female (A_2_B_1_), and sad female (A_2_B_2_). On a given trial, a participant is shown one of these faces, and must identify the face accordingly. **Figure 3A** illustrates a hypothetical GRT model for this example. In the figure, gender is perceptually separable from emotional expression, as indicated by the overlapping marginal distributions along the *y*-axis. On the other hand, emotional expression is not perceptually separable from gender, as the marginal distributions along the *x*-axis are not overlapping. There is a violation of PI for the sad-female stimulus (green distribution, showing a positive correlation), but not for the other stimuli. DS holds for emotional expression, as the bound used for this classification is orthogonal to the *y*-axis, but it does not hold for gender, as the bound used for this classification is not orthogonal to the *x*-axis.

Due to its simplicity, the 2 × 2 task is by far the most widely used design in the field. It requires presenting only four stimuli and measuring only four responses. Other designs, such as a 3 × 3 task (two dimensions, each with three levels), are also possible. However, these tasks are less practical because the considerable learning required by participants means that the experiment usually will require multiple experimental sessions (e.g., 5 days in Experiment 1 of Ashby and Lee, 1991). The working memory load of a 3 × 3 task is also taxing, as participants are required to remember nine unique stimuli and their unique responses. Because of these and other reasons (see Ashby and Soto, 2015; Soto et al., 2015), functions in the *grtools* package were developed specifically to deal with the 2 × 2 identification task.

The most common way to analyze the data from an identification task is through a *summary statistics* analysis, in which the researcher draws inferences about PI, PS, and DS by using summary statistics like proportion of correct responses, and measures of sensitivity and bias. An introductory tutorial about the exact statistics computed in this analysis can be found elsewhere (Ashby and Soto, 2015). For a rigorous treatment of the theory behind these analyses, see the following references: (Ashby and Townsend, 1986; Kadlec and Townsend, 1992a,b). Below, we focus only on how to obtain the results from summary statistics analyses using *grtools* and how they should be interpreted.

A second approach is *model-based analyses*, which consist of fitting one or more GRT models to the data obtained from an identification experiment. In traditional model-based analyses, several models are fit and then model selection approaches are used to determine which of those models provides the best account of the data (Ashby and Lee, 1991; Thomas, 2001). The models used implement different assumptions about PS, PI, and DS. Thus, selecting a particular model is equivalent to selecting those assumptions that explain the data best. An introductory tutorial to model fitting and selection with traditional GRT models of the 2 × 2 identification task can be found elsewhere (Ashby and Soto, 2015). Below, we focus on showing how to easily obtain the results from such a procedure using *grtools*.

Several problems have been identified with traditional GRT model-based analyses. The most important are that traditional analyses (1) do not allow a clear dissociation between PS and DS (Silbert and Thomas, 2013), (2) do not allow the full model to be fit to the data (because the full model has too many free parameters), and (3) are prone to over-fitting the data (Soto et al., 2015). All these problems are solved by the recently proposed General Recognition Theory with Individual Differences (GRT-wIND) model (Soto et al., 2015). GRT-wIND is fit simultaneously to the data from all participants in a particular experiment, because it assumes that some aspects of perception are common to all people. In particular, the model assumes that PS and PI either hold or do not hold across all participants, and that all participants perceive the same set of stimuli in a similar manner. Other aspects of the model are not common to all participants, but reflect individual differences. The model assumes individual differences in decisional strategies (e.g., in whether DS holds) and in the level to which people pay more or less attention to different dimensions. We recommend using GRT-wIND rather than traditional GRT models for the analysis of 2 × 2 identification experiments, as currently this is the only way to dissociate between perceptual and decisional processes in the 2 × 2 design.

### The 2 × 2 Garner Filtering Task

A task related to the 2 × 2 identification task is the 2 × 2 Garner filtering task (Garner, 1974; for a review, see Algom and Fitousi, 2016). The stimuli are identical to those used in the identification task, but the Garner filtering task asks participants to classify stimuli based on their value on a single dimension, while ignoring stimulus values on the second dimension. For example, a 2 × 2 Garner filtering task may require participants to classify faces according to their gender while ignoring their emotional expression (gender task), or to classify faces according to emotional expression while ignoring their gender (emotion task). This differs slightly yet significantly from the identification task; instead of identifying a unique stimulus, participants are now asked to group the stimuli based on a single dimension (e.g., gender or emotion).

**Figure 3B** illustrates a hypothetical GRT model for a Garner filtering task in which emotional expression is the relevant classification dimension, and gender is the irrelevant dimension. Assuming that the stimuli are the same as those from our previous example of the 2 × 2 identification task, the perceptual representations are also the same in this model. The main difference is in the decision bounds: rather than two bounds dividing space into four areas (one response per stimulus), there is now a single bound dividing space into only two areas (one response per expression level).

There are two important conditions in a Garner filtering task, that are presented to participants in separated experimental blocks. In baseline blocks, the value of the irrelevant dimension (the dimension that participants must ignore) is fixed across trials. For example, participants may have to classify faces according to their gender, while emotional expression is fixed at “happy.” In filtering blocks, the value of the irrelevant dimension is varied across trials. For example, participants may have to classify faces according to their gender, while emotional expression randomly varies between “happy” and “sad.”

If participants have difficulty separating one dimension from another, then a “Garner interference effect” (Garner, 1974) is expected: slower response times and lower accuracy are likely to occur on filtering blocks, compared to baseline blocks. Accuracy in the Garner filtering task is usually at ceiling levels, so most studies have measured the Garner interference effect in terms of response times.

Because response times are the most common dependent variable used for the filtering task, using GRT for data analysis requires making further assumptions about the way in which parameters in a GRT model are related to observed response time distributions. The simplest possible assumption is the *RT-distance hypothesis* (Murdock, 1985), which proposes that perceptual effects that are more distant from the decision bound produce faster responses. In **Figure 3B**, the representation for female/sad (green distribution) is closer to the bound than the representation for male/sad (orange distribution). This means that response times to classify sad faces should be slower when faces are female than when they are male. Ashby and Maddox (1994) showed that, if the RT-distance hypothesis is assumed, the data from a filtering task can be used to compute tests of dimensional separability that are *more diagnostic* than the Garner interference test.

In particular, whereas a violation of separability (perceptual or decisional) is likely to produce a Garner interference effect, a *context effect* is also likely to produce an interference effect (Ashby and Maddox, 1994). Context effects refer to the case in which the perception of a particular stimulus is changed depending on other stimuli presented closely in time. In the Garner task, such a change in context happens between the baseline (two stimuli are presented) and the filtering (four stimuli are presented) blocks. The effect of context on stimulus perception does not need to be related to separability at all. For example, variability in the irrelevant dimension during filtering blocks could produce spontaneous switches of attention toward that dimension, which are quickly remedied by switching attention back to the relevant dimension (see Yankouskaya et al., 2014). Such switches of attention would increase response times in the filtering blocks compared to the baseline blocks, even when dimensions are perceptually separable.

Ashby and Maddox (1994) proposed two tests of separability that are not affected by context effects: marginal response invariance (mRi) and marginal response time invariance (mRTi) tests. Context effects do not influence mRi and mRTi because these tests are computed from data originating from a single block type. Usually, the data should come from filtering blocks, because in these blocks all stimuli are presented at the same time, which reduces the likelihood of participants changing their response strategy depending on the specific stimuli presented.

The mRi test compares the proportion of correct responses for the relevant dimension across the two levels of the irrelevant dimension. If both PS and DS hold, then the mRi test should indicate no significant differences across levels of the irrelevant dimension. For example, mRi for gender across changes in emotion means that the probability of correctly classifying a face as male does not change depending on whether the face is sad or happy.

The mRTi test compares the full distribution of response times for correct classifications of the relevant dimension across the two levels of the irrelevant dimension. If both PS and DS hold, then the mRTi test should indicate no significant differences across levels of the irrelevant dimension. For example, mRTi for gender across changes in emotion means that the time it takes to correctly decide that a face is male does not change depending on whether the face is sad or happy. Because mRTi involves a comparison of whole response time distributions, it is the strongest test of separability available for the Garner filtering task (Ashby and Maddox, 1994).

As with traditional GRT analyses of the identification task, but unlike analyses based on GRT-wIND, the summary statistics analyses of the Garner interference task cannot determine whether violations of separability are due to perceptual or decisional factors. The usual approach is to assume that DS holds and make conclusions about PS. As indicated in the “Discussion” section below, there are ways to design the task so that DS is more likely to hold.

Although model-based analyses of data from the Garner filtering task are possible (Maddox and Ashby, 1996), they require previous knowledge about the perceptual distributions involved, which can be acquired by fitting a GRT model to the data from an identification task. That makes the analyses rather redundant, so applications of GRT to the filtering task have not used model-based analyses.

## Using *grtools*

### Installing *grtools*

The *grtools* package uses the computer programming language R (R Core Team, 2016). R is an open-source statistical software used by many researchers across a variety of scientific fields. R can be downloaded for free at http://cran.rstudio.com.

We highly recommend using RStudio to work with R. RStudio is an integrated development environment (IDE) for R, which provides a more user-friendly experience that includes a package manager, syntax-highlighting editor, tools for plotting, history, debugging, and workspace management. RStudio is also free and open-source, and it can be downloaded at https://www.rstudio.com/products/rstudio/download/.

The *grtools* package will require that you have a C++ compiler installed on your computer. For Windows users, the C++ compiler Rtools^1^ can be used. For Mac users, a C++ compiler can be installed through Xcode (found in the Apple Application Store). More instructions on how to install these compilers can be found in the *grtools* webpage: https://github.com/fsotoc/grtools.

To install *grtools*, start a session in R (or RStudio) and in the console type the following:


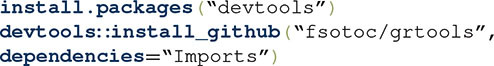


Now *grtools* is installed and available for use. To have access to *grtools* functions and analyses, you have to load the package into your current R session. Type the following in the console to load the *grtools* package:


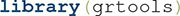


To access documentation that provides explanations and examples for the analyses in *grtools*, type the following in the console:


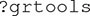


### Entering Data in R from a 2 × 2 Identification Task

Some data gathered through the 2 × 2 identification task must be excluded to allow accurate analyses using *grtools*. First, data from participants in the first few blocks of trials must usually be excluded. These data represent the participants’ learning periods for the task. GRT models only decisional and perceptual processes during steady-state performance; it is unable to model learning processes. A good approach is to set a learning criterion (e.g., a specific number of blocks finished, or a percentage of correct responses within the last *n* trials) and use data only after the criterion has been reached by a participant. Second, data from participants that perform at chance levels in the task should also be excluded. This is usually an indicator that the participant did not understand or learn the task well. Lastly, data from participants that perform near-perfectly should also be excluded. The GRT analyses included in *grtools* extract information about dimensional independence from the pattern of errors shown by participants. If there are only a few errors, *grtools* analyses will be misleading and inaccurate. This requirement is common in psychophysics, as other tools like signal detection theory and classification image techniques also work only when errors are made.

Because some stimuli (e.g., faces) are very easy to identify, obtaining identification errors might require manipulating the stimuli to increase task difficulty. Manipulations that increase errors include decreasing image contrast, decreasing presentation times, and increasing stimulus similarity through morphing and other image manipulation techniques.

The data from an identification experiment are summarized in a confusion matrix, which contains a row for each stimulus and a column for each response. In the 2 × 2 design, there are four stimuli and four responses, resulting in a 4 × 4 confusion matrix with a total of 16 data elements for each test participant. An example from our hypothetical experiment dealing with face gender and emotion is shown in **Table 1**. The entry in each cell of this matrix represents the number of trials in which a particular stimulus (row) was presented and a particular response (column) was given. Thus, entries on the main diagonal represent the frequency of each correct response and off-diagonal entries describe the various errors (or confusions). For example, the number in the top-left in **Table 1** represents the number of trials on which stimulus A_1_B_1_ was presented and correctly identified (140 trials). The next cell to the right shows the number of trials on which stimulus A_1_B_1_ was presented, but the participant incorrectly reported seeing stimulus A_2_B_1_ (36 trials). Each row of values must sum up to the total number of trials on which the corresponding stimulus was presented.

**Table 1:** Data from a simulated identification experiment with four face stimuli, created by factorially combining two levels of emotion (happy and sad) and two levels of gender (male and female).

	Response			
Stimulus	Happy/Male	Sad/Male	Happy/Female	Sad/Female
Happy/Male	140	36	34	40
Sad/Male	89	91	4	66
Happy/Female	85	5	90	70
Sad/Female	20	59	8	163

When entering the data to R, be careful to order rows and columns in the same way as shown in **Table 1**; that is, beginning with the first row/column and ending with the last row/column, the correct order is: A_1_B_1_, A_2_B_1_, A_1_B_2_, and A_2_B_2_.

There are at least two ways in which the data from a confusion matrix can be entered into R for analysis with *grtools*. The first option is to directly enter the data as an R object in the matrix class. This can be done through the R function 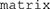, as in the following example:


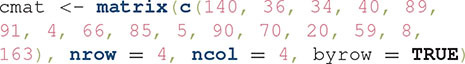


Note that we have entered the data as a single vector inside the function 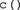, starting with the data from the first row, then the second row, and so on. The function 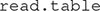 is then used to shape this vector into an actual confusion matrix.

The second option is to input the confusion matrix into a spreadsheet application, such as Microsoft Excel. As before, the data must be ordered into a 4 × 4 matrix, as shown in **Table 1**. After entering the data, go to the “File” menu and choose “Save as…”. In the drop-down menu titled “Format” choose “Comma Separated Values (.csv).” Name your file, choose the folder where you want to store it, and click “Save.” In our example, we will name the file “data.csv” and store it in the directory “home/Documents.”

To import the data to R, make sure first that your working directory is the folder that contains your data file. This is easy to do in RStudio, where you can simply navigate through your folders using the “Files” panel at the bottom-right of the screen. Once you get to your destination folder, click in the “More” menu of the “Files” panel, and choose “Set as Working Directory.”

Read the file using the function 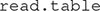, as in the following example:


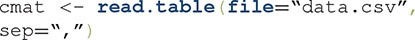


Here, the 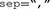 argument specifies the separator used in the data file, which is a comma. Alternatively, if you know the full path of your file, you can include it in the 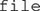 argument (e.g., 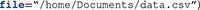) and skip the step of changing the working directory.

The data table is now available as a data frame named 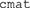. The final step is to convert the data into a matrix object, using the 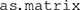 function. The following command reassigns 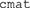 as a matrix:


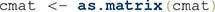


### Summary Statistics Analysis of the 2 × 2 Identification Task

There are two types of summary statistics analyses that can be performed for the 2 × 2 identification design: macro- and micro-analyses (Kadlec and Townsend, 1992a,b). Both depend on the computation of only three statistics–proportion of correct responses, sensitivity, and bias– for different combinations of stimuli and responses. The main difference is that macro-analyses compare statistics linked to different stimuli in the task, whereas micro-analyses compare statistics linked to the same stimulus in the task.

Both the computation of sensitivity measures and the statistical tests for proportions require that proportions are larger than zero and smaller than one. For this reason, *grtools* replaces zeros and ones with values arbitrarily close to them (zero is replaced with 10^-10^, one with 1–10^-10^) and issues a warning in the R console.

A complete set of macro-analyses can be performed in *grtools* with only three lines of code executed in the R console. The first line performs the actual analysis, using the data that we previously stored in the matrix named 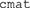:


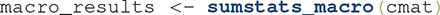


This stores the results in an object named 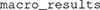, of class 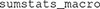. Our second line of code allows us to see a summary of the results:


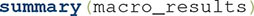


**FIGURE 4:**
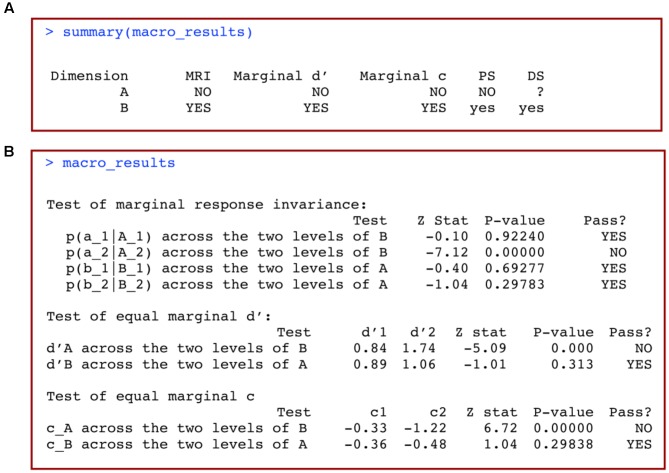
**(A)** Summary and **(B)** full results of a macro-analysis of data from a 2 × 2 identification design with *grtools*.

This should produce an output table like the one reproduced in **Figure 4A**. The interpretation of this table is straightforward. Each row represents a particular dimension, and the columns titled “MRI,” “Marginal d′” and “Marginal c” include information about whether or not each of these conditions holds according to the statistical tests performed (see Ashby and Soto, 2015). Only two values will be displayed in these columns: “YES” to indicate that the condition holds, and “NO” to indicate that the condition does not hold (i.e., a significant failure was detected in the relevant test).

Information about the analyses’ conclusions is stored in the final two columns, named “PS” for perceptual separability, and “DS” for decisional separability. The results in these columns are formatted similarly to the results from Kadlec’s classic msda2 software (Kadlec, 1995; **Table 1**):

• yes means that PS/DS may hold (weak evidence).
• NO means that PS/DS does not hold.
• ? yes means that PS/DS is unknown but possibly yes
• ? no means that PS/DS is unknown but possibly no
• ? means that PS/DS is unknown

In our specific example (**Figure 4A**), we conclude that dimension A is not perceptually separable from dimension B (NO), but dimension B may be perceptually separable from dimension A (yes). We also conclude that dimension B may be decisionally separable from dimension A (yes), but there is not enough information to reach a conclusion about whether dimension A is decisionally separable from dimension B (?).

If we want to obtain more specific information about the tests performed in the macro-analysis, we simply type the name of the object in which we stored our results:


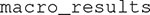


This produces the output in **Figure 4B**. Results from each of the tests summarized in **Figure 4A** are now displayed in full detail, including the specific subtests, computed statistics, *p*-values and conclusions.

Micro-analyses are another method of summary statistic analyses available in *grtools* (see Ashby and Soto, 2015). These analyses use a different set of summary statistics to test assumptions about both PI and PS. The sequence of commands required to perform a full set of micro-analyses is similar to the macro-analyses described above. The first line performs the micro-analyses using the data stored in the matrix 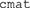:


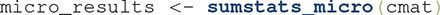


This stores the results in an object named 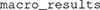. To see a summary of the results, you can use this 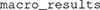 object as input to the 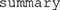 function:


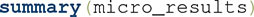


**FIGURE 5:**
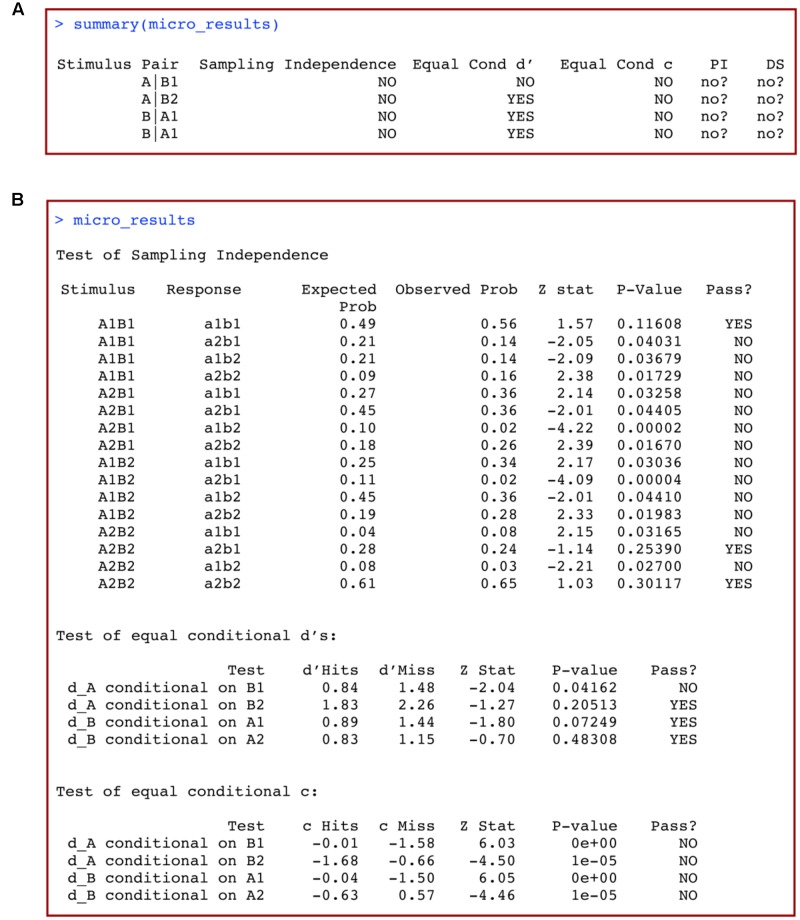
**(A)** Summary and **(B)** full results of a macro-analysis of data from a 2 × 2 identification design with *grtools*.

This produces an output table like the one reproduced in **Figure 5A**. Each row represents a unique stimulus, and the columns titled “Sampling Independence,” “Equal Cond d’” (Equal Conditional d’), “Equal Cond c” (Equal Conditional c), include information about whether or not each of these conditions holds according to the statistical tests performed (Ashby and Soto, 2015). Conclusions regarding PI and DS are stored in the final two columns, labeled PI and DS, respectively. The format of the results is the same as for the macro-analyses. In our example (**Figure 5A**), both PI and DS are unknown, but possibly do not hold.

If you want more specific information about the tests conducted, simply type the name of the object previously used to store the results of this analysis:


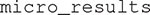


This produces the output in **Figure 5B**. Results from each one of the tests summarized in **Figure 5A** are now displayed in full detail, including the specific subtests, computed statistics, *p*-values, and conclusions.

Recent research has found that summary statistic analyses of the 2 × 2 identification design can sometimes lead to wrong conclusions about separability and independence (Mack et al., 2011; Silbert and Thomas, 2013). For this reason, it is good practice to perform the additional model-based analysis of the data that is described in the following two sections.

### Model-Based Analyses of the 2 × 2 Identification Task Using Traditional GRT Models

Model-based GRT analyses have been used less often than summary statistics in the literature, probably due to the fact that implementing a variety of models, fitting them to data, and selecting among them requires quantitative skill and a deep understanding of the theory. One of the goals of developing *grtools* was to provide experimental psychologists who lack formal quantitative training with tools to easily perform and interpret model-based analyses. Thus, we wrote the software placing ease-of-use above flexibility: a full model-based analysis can be performed with only three lines of code, but the researcher is not free to choose what models to test or the procedures used for model fit and selection^2^. We start by briefly reviewing our choices regarding these points.

Because past research almost exclusively has used the 2 × 2 identification task (for an exception, see Ashby and Lee, 1991), *grtools* includes functions to model data only from that task. The design is popular because it has a number of advantages: it requires a relatively short experiment, it does not tax the participants’ working memory as larger designs do, and it allows a study of stimulus “components” that cannot be ordered along a continuous dimension (e.g., face identity; see Soto et al., 2015).

Our focus on the 2 × 2 identification task means that DS must be assumed throughout the analysis and cannot be tested. The reason is that it has been shown that it is impossible to perform a valid test of DS using traditional modeling of the 2 × 2 identification task (Silbert and Thomas, 2013). Additionally, the 2 × 2 identification task generates only 16 data points per participant. Due to this small sample size, complex models (i.e., models with many parameters) cannot be adequately fit, and additional assumptions must be made to simplify all models. As in previous related research, the model-based analysis implemented in *grtools* assumes that all variances of the four perceptual distributions are equal to one. All of these problems are solved by analyses using GRT-wIND (see Soto et al., 2015), which we cover in the next section.

**FIGURE 6:**
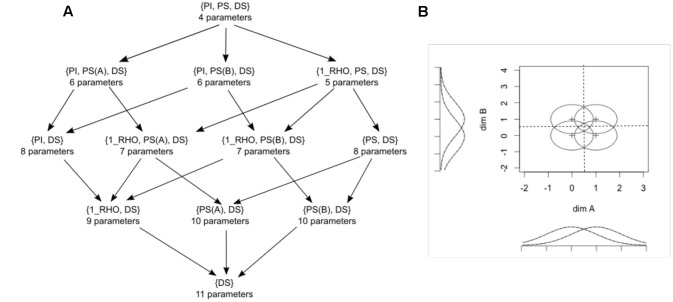
**(A)** Hierarchy of models used in a model-based analysis of data from a 2 × 2 identification task using traditional general recognition theory with *grtools.* PI stands for perceptual independence, PS for perceptual separability, DS for decisional separability, and 1_RHO for a single correlation in all distributions. The number of free parameters in each model is indicated below its label. **(B)** Initial configuration assuming PS, PI, and DS.

**Figure 6A** shows the 12 models used in the *grtools* analysis, ordered in a hierarchy in which models that make more assumptions are placed higher, and models that make fewer assumptions are placed progressively lower. This hierarchy has been modified from one presented earlier by Ashby and Soto (2015), which in turn was based on an earlier hierarchy by Thomas (2001).

In **Figure 6A**, DS stands for decisional separability. As it can be seen, this property is assumed in all models. PS stands for perceptual separability and PI stands for perceptual independence. 1_RHO stands for a specific violation of PI in which the correlation (i.e., which measures the strength of the PI violation) is the same for all four perceptual distributions. At the top of the hierarchy in **Figure 6A** is the most constrained model, which assumes PI and PS in addition to DS. This model has a total of four parameters that can be varied to provide a good fit to the data (so there are four “free parameters”). Each of the models one step lower in the hierarchy relaxes a single assumption of the more general model. Relaxing an assumption requires adding one or more free parameters to the model. The process of relaxing assumptions by adding parameters continues until we arrive at the least constrained model at the bottom, which assumes only DS.

In past applications (e.g., Ashby and Lee, 1991; Thomas, 2001; Soto et al., 2015) model selection proceeded by a series of likelihood ratio tests comparing models joined by arrows in **Figure 6A** (for details, see Ashby and Soto, 2015). The use of the likelihood ratio tests allowed the researcher to fit only some of the models in the hierarchy to the data, speeding up the analysis. However, the increase in speed came at the cost of consistency in the model-selection procedure: in most cases, the likelihood ratio tests could not decide between several candidate models, which then had to be compared by some other criterion. Fortunately, this is no longer necessary, as the *grtools* code is fast enough^3^ that the process of fitting the whole hierarchy to the data of a participant and selecting the best model can be executed in about 20 s. This is done through a single line of code:


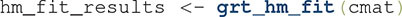


Here we have used again the data previously stored in the matrix 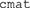. What the function 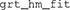 does in the background is fit all the models in **Figure 6** to the data in 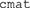, using maximum likelihood estimation (see Myung, 2003) through the R function 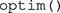. The likelihood function of a GRT model may have several “hills” and “valleys.” Ideally, the optimization algorithm would only stop at the top of the highest hill (the maximum likelihood), but it is also possible for the algorithm to stop at one of the smaller hills, which is known as a local maximum (see Myung, 2003; Ashby and Soto, 2015). To avoid this problem, the search for the best set of parameters for a particular model is performed 10 times, each time starting from a different set of initial parameter values. The best solution is kept from these 10 runs of the algorithm.

Each set of initial parameters is determined by adding a random perturbation to a fixed configuration, shown in **Figure 6B**. The maximum value of the random perturbation for each parameter is set by default to 0.3.

Although the default settings for 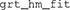 have worked well in our own research, users can change all of them. For example, to fit each model 20 times and use a maximum random perturbation of 0.5, one can use the following command:


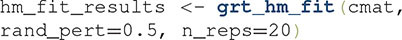


Furthermore, users can have additional control over the optimization process by passing a list of control parameters as final argument to 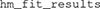, in the same way in which this is usually done for the optim function [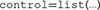; for more information, type 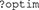 in the R console].

After finding maximum likelihood estimates for all models, the best model is selected by computing the (corrected) Akaike information criterion (AIC) of each model (Bozdogan, 2000; Wagenmakers and Farrell, 2004; for a tutorial on its application to GRT, see Ashby and Soto, 2015). A summary of the results of the model fitting and selection procedures can be obtained through the following command:


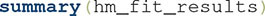


**FIGURE 7:**
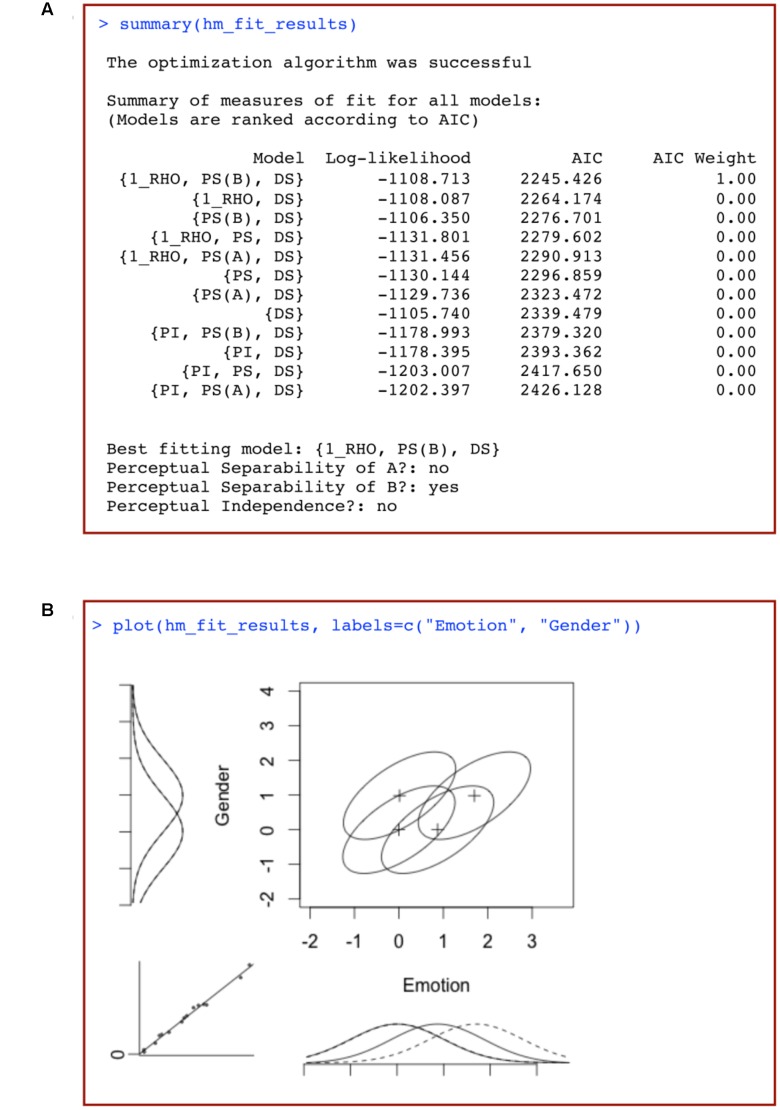
**(A)** Summary of the results of a model-based analysis of data from a 2 × 2 identification task using traditional general recognition theory with *grtools*, and **(B)** plot of the best-fitting model resulting from such analysis, where marginal distributions for a given dimension are plotted using either solid or dotted lines depending on the level of the opposite dimension, and the bottom-left insert is a plot of the observed response proportions against those predicted by the model.

This should produce an output similar to that shown in **Figure 7A**. The first column of **Figure 7A** lists the different models tested, ordered from best to worst fit to the data. 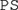 stands for perceptual separability, 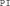 for perceptual independence, 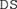 for decisional separability, and 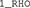 describes a model with a single correlation parameter for all distributions. In our example, the model that fit the data best is summarized as 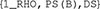. This is a model that assumes a single correlation parameter (and so a violation of perceptual independence), assumes PS for dimension B, and assumes DS.

The following two columns list the log-likelihood and AIC values for each of the models fitted to data. These values are used to assess which models provide the best description of the observed data. In the table produced by *grtools*, models are ranked based on the AIC value, which takes into account both the model’s fit to data as well as the model’s complexity. A relatively low AIC value represents a model that provides a good description of the data without being overly complex and flexible (see Bozdogan, 2000; Wagenmakers and Farrell, 2004). The final column represents the AIC weights (see Wagenmakers and Farrell, 2004), which can be interpreted as the probability that a given model is closest to the true model among those tested. In the case in **Figure 7A**, the probability of model 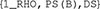 being closest to the true model is so high that it receives an AIC weight of one after rounding, with all other models receiving a weight of 0. Thus, in this case we can be very confident that model 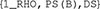 provides the best account of the data of all the models that we tested.

Underneath the data table, the code also provides a summary table that lists the best-fitting model, and whether PS of A, PS of B, and PI were violated.

To help visualize what the best-fit model looks like, *grtools* provides aid through the 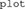 function:


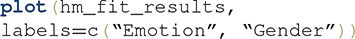


This produces a graphical representation of the best-fitting model, like the one shown in **Figure 7B**. The elements in this figure should be interpreted as explained earlier: each ellipse represents a single Gaussian distribution, with the plus sign representing its mean. The tilt of the ellipse represents the correlation between the perceptual effects in the two dimensions. Any tilt is indicative of a failure of PI. It can be seen that the distributions show a positive correlation (represented by 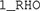 in the best-fitting model’s label), indicative of a failure of PI.

Marginal distributions are plotted to the left and bottom of the figure. The marginal distributions for a given dimension are plotted using either solid or dotted lines depending on the level of the opposite dimension. These provide a visual test of whether the data suggest violations of PS: failures of PS are suggested by non-overlapping solid and dotted marginal distributions. In **Figure 7B**, the non-overlapping marginal distributions along the expression dimension suggest a failure of PS of expression from gender [which is why 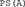 is not included in the best-fitting model’s label]. The overlapping marginal distributions along the gender dimension suggest PS of gender from expression [which is why 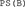 is included in the best-fitting model’s label].

The insert at the bottom-left shows a plot of the observed response proportions against the response probabilities predicted by the best-fitting model. This plot allows a visual evaluation of how well the model fit the data. In a perfect fit, all the dots would land on the diagonal.

### Model-Based Analyses of the 2 × 2 Identification Task Using GRT-wIND

Unlike the other analyses that we have illustrated, GRT-wIND accounts for the data from all participants in an experimental group who completed the 2 × 2 identification task. Suppose we have data from five participants that we have collected in five confusion matrices, one for each participant. The first step in applying GRT-wIND is to take all of our confusion matrices and concatenate them in a list:


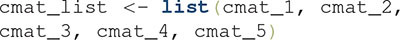


An example of properly formatted data is included with *grtools*, and can be accessed by typing the following in the R console:


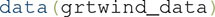


**FIGURE 8:**
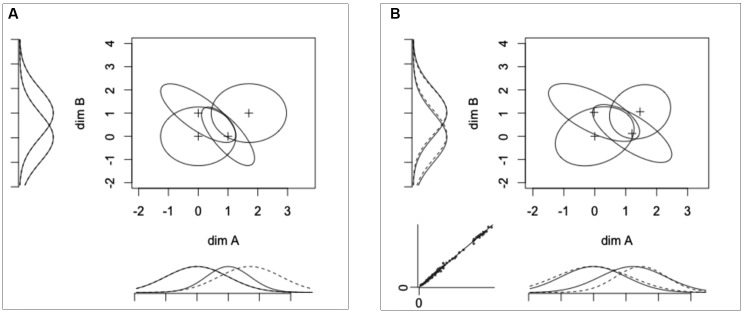
**(A)** Original GRT-wIND model used to generate the data in our example (see main text) and **(B)** model recovered by *grtools*, where marginal distributions for a given dimension are plotted using either solid or dotted lines depending on the level of the opposite dimension, and the bottom-left insert is a plot of the observed response proportions against those predicted by the model.

You can look at the loaded data by typing 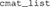 in the R console. These data have been sampled directly from the model shown in **Figure 8A**. This model was built to show PS of dimension B from dimension A, but failure of PS of dimension A from dimension B (marginal distributions along the *x*-axis are not aligned, see **Figure 8A**). There are also violations of PI (i.e., negative correlations) for stimuli A_2_B_1_ and A_1_B_2_. Furthermore, the decision strategies of hypothetical participants randomly deviated from DS on both dimensions.

A model-based GRT-wIND analysis starts by fitting the full model to the data in 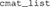, using the following command:


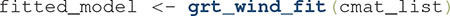


This line of code will fit a full GRT-wIND model to the experimental data, using maximum likelihood estimation (see Soto et al., 2015). By default, the algorithm starts with a set of parameters reflecting PS, PI, and DS (see **Figure 6B**), which are slightly changed by adding or subtracting a random value. The algorithm then efficiently searches the parameter space to determine the parameter values that maximize the likelihood of the data given the model. As indicated above, to avoid finding a local maximum, one solution is to fit the model to data several times, each time with a different set of starting parameters. This procedure is time consuming, but it provides more valid results, so we recommend using it. *grtools* includes a special function to perform such multiple fits to a GRT-wIND model:


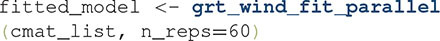



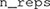
 must be provided and it represents the number of times that the model will be fit to data. In our own practice, we have chosen a rather high value of 60 for this parameter (Soto and Ashby, 2015; Soto et al., 2015). Good results (i.e., recovery of the true model producing the data) can be sometimes obtained with 20–30 repetitions. Future simulation work will be necessary to determine the minimum number of repetitions that produces good results when fitting GRT-wIND across a variety of circumstances. In the meanwhile, our recommendation is to proceed with caution and use a high value for 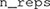. This will considerably increase the time that the analysis will take to finish, but it will ensure more valid results.

The function 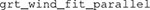 takes advantage of multiple processing cores in the computer when these are available. By default, the function will use all the available cores minus one. This means that using a machine with multiple cores will considerably speed up the analysis.

The default settings for 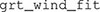 and 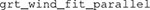 have worked well for us, and we recommend that researchers with limited modeling experience use them. However, options to control the optimization algorithm and starting parameters are available for both functions. Interested researchers should read the documentation for each function, which is available by executing the commands 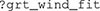 or 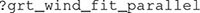 in the R console.

To visualize the best-fitting model found by the GRT-wIND analyses, you can call the 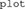 function:


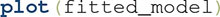


This produces **Figure 8B**, a representation of the recovered model that has nearly the same features as the true model shown in **Figure 8A**, and can be interpreted in the same way.

Additionally, you can run statistical tests to determine whether PS, DS, or PI were violated. Currently, *grtools* offers two ways to perform such statistical tests: Wald tests (see Soto et al., 2015) and likelihood ratio tests (see Ashby and Soto, 2015).

The Wald tests have the advantage of being relatively fast to compute. However, they require an estimate of the Hessian matrix of the likelihood function associated with the maximum likelihood estimates (see appendix of Soto et al., 2015), which is computationally costly and therefore may take several minutes. Even so, once the Hessian is obtained, all tests can be computed in seconds.

Unfortunately, some estimates of the Hessian are not useful for the computation of a Wald test (i.e., when the Hessian is not positive definite, see Gill and King, 2004). In our experience, the estimates provided by R (e.g., through the package “numDeriv,” see Gilbert and Varadhan, 2015) are often problematic. We are currently working on implementing procedures to estimate the Hessian that have proven more successful in our experience (e.g., DERIVEST; D’Errico, 2006).

Because of these problems with the Wald test, we recommend researchers perform likelihood ratio tests instead. These tests are slow to compute, but they do not require numerical estimation of the Hessian. A full series of likelihood ratio tests for PS, PI, and DS is performed by using the following line of code:


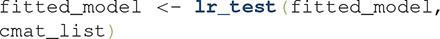


Each likelihood ratio test requires refitting GRT-wIND to the data an additional time, except this time with a model in which the assumption being tested (e.g., PS of dimension A) is assumed to hold. As before, this process is repeated a number of times with different starting parameters, obtained by adding random perturbations to the parameters estimated earlier with 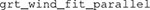. Because these parameters are likely to be a better starting guess than the model in **Figure 6B**, for most cases we can obtain good results without having to run a high number of repetitions. By default, the function 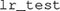 runs the optimization algorithm 20 times for each test. This value can be changed by explicitly setting 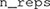 to a different value, as you did in the call to 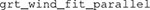.

**FIGURE 9:**
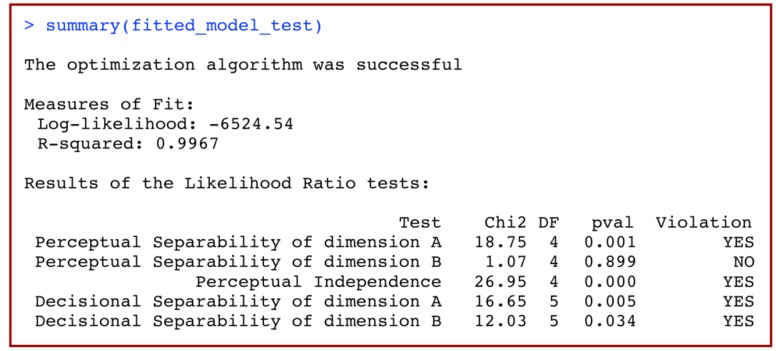
**Summary of the results of a model-based analysis of data from a 2 × 2 identification task using general recognition theory with individual differences (GRT-wIND)**.

Using 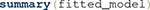 should now print to screen an output similar to that shown in **Figure 9**. There are three parts to this output. The first line is a message indicating whether the optimization algorithm was successful in finding the maximum likelihood estimate of the GRT-wIND parameters. Any problem in the optimization process (which may invalidate the results of this analysis) will be described in this line. This is followed by a summary of the fit of the full model to data, including both the obtained log-likelihood and R-squared (the proportion of the variance in the data explained by the model) of the best-fitting model. The third part of this summary output includes the results for all likelihood ratio tests, in a table with columns including a description of the test, the Chi-squared test statistic, degrees of freedom, *p*-value, and the test’s conclusion (i.e., whether or not PS, PI or DS is violated). In our example, the likelihood ratio tests accurately conclude that PS holds for dimension B (i.e., NO violation) but fails for dimension A (YES violation), that PI does not hold (YES violation), and that DS does not hold for either dimension (YES violation).

If only some likelihood ratio tests are of interest, it is possible to explicitly indicate what tests to run:


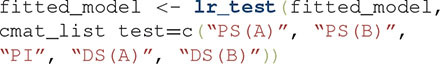


To run only some of these tests, simply keep the strings in the test array that correspond to a test that you want to run, and delete other strings. For example, to run tests of PS and DS of dimension A only, you should use


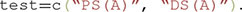


### Entering Data in R from a 2 × 2 Garner Filtering Task

Researchers should consider excluding some data from a Garner filtering task to allow accurate analyses using *grtools*. As with the identification ask, initial practice trials–used to teach participants the mapping between levels of the relevant dimension and response keys–should not be included in the analysis, and data from participants that perform at chance levels in the task should also be excluded. Unlike analysis of the identification task, researchers might choose to include the data from participants that perform near-perfectly. If such participants are included in the analysis, then all conclusions should focus on the analysis of response time data. Tests based on the analysis of proportion of correct choices (the Garner interference test on proportion of correct responses and the MRI test) would be invalid and should be ignored.

The statistical tests for proportions require that proportions are larger than zero and smaller than one. For this reason, *grtools* replaces zeros and ones with values arbitrarily close to them (zero is replaced with 10^-10^, one with 1–10^-10^) and issues a warning in the R console.

*grtools* includes a routine to perform all the summary statistic analyses for the 2 × 2 Garner filtering task described above using only a couple of commands. Data are analyzed for each participant individually, and the first step in the analysis is to create a data frame with the participant’s data. Each row in the data frame includes data from one trial, with the following column organization (for a description of the task, see section “The 2 × 2 Garner Filtering Task”):

• Column 1: Block type, with a value of 1 for baseline blocks and a value of 2 for filtering blocks
• Column 2: Level of the relevant dimension, with values 1 and 2
• Column 3: Level of the irrelevant dimension, with values 1 and 2
• Column 4: Accuracy, with a value of 0 for incorrect trials and 1 for correct trials
• Column 5: Response time

The data can be entered into a data spreadsheet processor like Microsoft Excel, saved as a comma-separated file, and then imported to R using the function 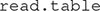, as explained for 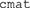 above. For this analysis, however, the imported data should not be converted to a matrix. An example of properly formatted data is included with *grtools*, and can be accessed by typing the following in the R console:


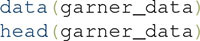


The first line of code loads the 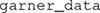 data frame into our current R session, and the second line of code prints the column names and the first few rows on the console.

### Summary Statistics Analysis of the 2 × 2 Garner Filtering Task

We perform the summary statistic analysis on these data using the following line of code:


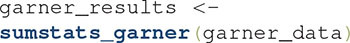


The following line of code provides a comprehensive summary of the results:


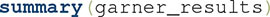


**FIGURE 10:**
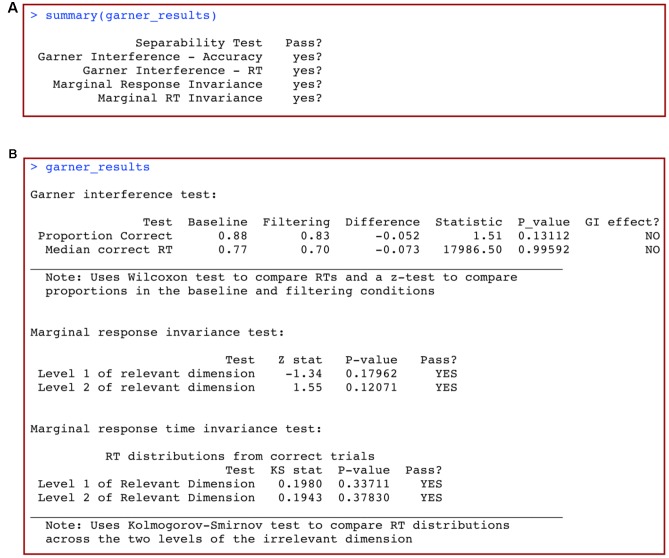
**(A)** Summary and **(B)** full results of a summary statistics analysis of data from a 2 × 2 Garner filtering task with *grtools*.

This produces the output shown in **Figure 10A**. The first column lists the name of the separability test, while the second column shows whether the test was passed (yes?) or not (NO) according to the analysis. The statistical test used by default to compare response time distributions in the mRTi test is the well-known Kolmogorov–Smirnov test, or KS test. If the conclusions from these analyses are that all tests are passed (yes?), then this suggests that the relevant dimension might be separable from the irrelevant dimension. If any of the conclusions from these analyses indicate that the test is not passed (NO), then this suggests that the relevant dimension is not separable from the irrelevant dimension.

To view a more detailed description of the results of this analysis, simply call the R object in which you stored the results:


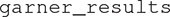


This produces the output shown in **Figure 10B**. Full details and information of the analysis are shown, including the specific subtests, computed statistics, *p*-values, and conclusions.

## Discussion

We have described an R package with functions that perform a variety of statistical analyses to determine independence and separability of perceptual dimensions according to GRT. Our package focuses heavily on analyses of the 2 × 2 design, which is easier to implement and can be applied more broadly than larger designs. Still, we expect that future releases of *grtools* will include analyses for larger designs, as well as better algorithms for the estimation of the Hessian in Wald tests (Soto et al., 2015), tests to compare the results of two groups (Soto and Ashby, 2015), and model-based analyses of response time data (Maddox and Ashby, 1996).

Before concluding, we would like to provide some general recommendations regarding design and analyses aimed at determining dimensional independence and separability:

1. *For best results, run a 2 × 2 identification experiment and analyze it using GRT-wIND.* The 2* ×* 2 identification task is appealing due to its simplicity and because it is easy to run. Currently, model-based analysis with GRT-wIND is the only way to dissociate perceptual and decisional processes in the 2 × 2 identification task. Although the Garner filtering task is very popular among researchers, this task does not allow one to dissociate perceptual and decisional types of separability, as does the identification task.
2. *Calibrate stimulus values in the identification task to ensure that all participants produce a moderate rate of errors.* Participants that show perfect performance or performance near chance levels contribute no information to the analysis. For this reason, you should calibrate your stimuli to make sure that participants show a moderate error rate (∼25–35%). Common ways in which this can be achieved are by changing presentation times, contrast levels, and morphing levels (for faces and other objects). Usually, running some pilot participants is enough to settle on a particular set of stimuli. One possibility is using adaptive procedures (for reviews, see Leek, 2001; Lu and Dosher, 2013) to bring the error rates of all participants to the same level, by varying some stimulus feature that is unrelated to the dimensions under study.
3. *If you have the resources, use a concurrent operations approach by also running a 2 × 2 Garner filtering task*. In theory, the results from this second task cannot give more information about dimensional independence than the results from the identification task. In practice, it is advantageous to verify that the results obtained with the identification task, and analyses based mostly on choice proportions, are in line with results obtained with the Garner filtering task and analyses based mostly on response times.
4. *Use instructions that will increase the likelihood of DS*. Many of the analyses described here are only valid if DS holds. Fortunately, experimental results have shown that there are ways to encourage participants to use such decisional strategies, such as providing instructions indicating that stimuli follow a grid configuration (e.g., a 2 × 2 grid) and spatially positioning response buttons in that configuration (Ashby et al., 2001).
5. *Use all available tests of separability for the Garner filtering task.* The most common test of separability for the Garner task is the Garner interference test. As we have seen before, two other tests of separability are available –mRi and mRTi– and both are more diagnostic about dimensional separability than the Garner interference test. Given that there is no additional cost to perform such tests, we believe that most researchers do not perform them because they simply do not know how. Fortunately, *grtools* allows all three tests to be performed using a single command.
6. *Include a control group that provides a benchmark of separability*. Violations of separability are common, and features of your task and design might produce such violations. Factors such as training in a categorization task can influence PS as well (Soto and Ashby, 2015). For these reasons, it is a good idea to include a group that would serve as a benchmark, if the design does not already involve more than one group. That benchmark could be very simple; examples include a group presented with dimensions known to be separable (e.g., orientation and width of gratings, shape, and color) or integral (e.g., brightness and saturation; unfamiliar face identities).
7. *Make sure that your results are generalizable, rather than explainable by task features*. The simplicity of the 2 × 2 tasks comes at a cost: only four stimuli are studied, although the number of combinations of levels of the two dimensions under study could be infinite. You should be careful to not over generalize when interpreting the results from a single set of stimuli. Ideally, several experiments should be performed before reaching a conclusion, perhaps parametrically varying task factors such as difficulty (Yankouskaya et al., 2014). A good and low-cost way of learning whether task features can explain performance in a visual task is by analyzing the performance of an ideal observer in the task (for a GRT analysis of an ideal observer’s performance, see Experiment 2 of Soto and Wasserman, 2011).

In the past, GRT has been popular among mathematical psychologists who have the technical knowledge to implement statistical analyses using it. Most of the research using GRT outside this community was facilitated by Kadlec’s publication of the *mdsda* program (Kadlec, 1995, 1999), which performed summary statistics analyses of data from the identification tasks. Recently, another R package has been made public that allows data analyses using traditional GRT models (Silbert and Hawkins, 2016). However, this package requires a more active involvement from the researcher during the analysis. The researcher must have a good understanding of the relation between a variety of tests and the concepts of PI, PS, and DS. For model-based analyses, the researcher must decide what models to fit and compare, and make a selection based on measures of fit. On the other hand, *grtools* was developed with the typical experimental psychologist in mind, someone who wants to make use of the sophisticated analytical tools offered by GRT, but does not have the training to implement the analyses, make critical decisions about procedures, and interpret the overall pattern of results. Our package allows researchers to perform full GRT analyses with only three commands, and explore the results in a way that highlights the most important conclusions from the study. Importantly, *grtools* is the only currently available package implementing summary statistics analyses of the widely used Garner filtering task and model-based analyses with GRT-wIND (Soto et al., 2015). The latter are the only analyses capable of dissociating perceptual and decisional forms of separability with a 2 × 2 identification design. We hope that the availability of *grtools* will lead to a wider application of GRT to the analysis of dimensional interactions.

## Author Contributions

FS programmed the software described in this article, created examples and figures, and wrote the article. EZ programmed the software described in this article. JF programmed the software described in this article. FA wrote the article and supervised the project.

## Conflict of Interest Statement

The authors declare that the research was conducted in the absence of any commercial or financial relationships that could be construed as a potential conflict of interest.

## Footnotes

1 http://cran.r-project.org/bin/windows/Rtools/

2 However, since our software is open-source and distributed under a creative commons license, any researcher can modify the original code to fit his/her own needs.

3 The code in *grtools* is relatively fast for two reasons: (1) we use an efficient method of numerical integration proposed by Ennis and Ashby (2003) to compute the likelihood of the data for a given GRT model, and (2) we implement this method through a C++ function, which is called from R using the package Rcpp (Eddelbuettel et al., 2011).
